# A booklet self-help intervention to reduce depressive symptoms among people living with HIV in Botswana: study protocol for a randomized controlled trial

**DOI:** 10.1186/s13063-019-3584-0

**Published:** 2019-08-09

**Authors:** Boitumelo Vavani, Vivian Kraaij, Phillip Spinhoven, Nadia Garnefski

**Affiliations:** 1 Department of Psychology, University of Botswana, P/Bag UB00705, Gaborone, Botswana; 2 Leiden University, PO Box 9500, 2300 RB Leiden, Netherlands

**Keywords:** HIV, Depression, Self-help, Cognitive behavioral therapy, Coaching, Randomized controlled trial, Africa, Botswana

## Abstract

**Background:**

The treatment of mental health issues among people living with HIV (PLH) in Botswana is yet to be addressed. A recent study revealed that depressive symptoms are highly prevalent in a sample of PLH in Botswana. Based on empirical findings of a study that investigated intervention targets for PLH in Botswana, a self-help program with coaching in booklet format in the Setswana and English languages was developed, composed of cognitive behavioral techniques, coping skills interventions, and goal adjustment training. We will investigate the program for effectiveness in the treatment of depressive symptoms among PLH. Additionally, we will investigate treatment moderators and mediators. This paper describes the study protocol.

**Methods/design:**

A randomized controlled trial will be conducted to compare the booklet self-help program with coaching with an attention-only control condition, by including pre-test, post-test, and follow-up assessments. We aim to enroll 200 participants with mild to moderate depressive symptoms into the study. The self-help program contains the following main components: activation, relaxation, changing maladaptive cognitions, and the attainment of new personal goals. This content is covered over six lessons to be completed in a maximum of 8 weeks. It uses a combination of psycho-education, assignments, and exercises. The participants will work on the program 1–2 h every week for 6 weeks (maximum 8 weeks). Coaches will offer support and motivate the participants. For both groups, depressive symptoms and possible mediators will be measured three times during the intervention, and at pre-test, post-test, and follow-up.

**Discussion:**

If the intervention is found to effectively treat depressive symptoms, it will be implemented and thus help improve the psychological health of PLH in Botswana.

**Trial registration:**

Netherlands Trial Register, NTR7428. Registered on 23 August 2018.

**Electronic supplementary material:**

The online version of this article (10.1186/s13063-019-3584-0) contains supplementary material, which is available to authorized users.

## Background and objectives

In sub-Saharan Africa, the rate of new HIV infections as well as mortality due to HIV has reportedly gone down in recent years [1]. The decrease in HIV mortality and new infections can be credited to an increased access to antiretroviral therapy (ART) as well as the introduction of other HIV prevention programs such as prevention of mother-to-child transmission (PMTCT) and increased access to condoms [1]. Consequently, HIV is now considered a chronic illness [2–4], and this shift has led to increased attention to the overall management of HIV. There have been great investments in policies and medical programs that ensure easy access to ART by people living with HIV (PLH) [5]. This has been the case particularly in Botswana. The UNAIDS 2017 report [1] shows an overall increase in ART access by PLH in Botswana and sub-Saharan Africa.

While medical care has received a lot of attention, addressing the mental health care of PLH has lagged behind, despite the reported high prevalence of mental health problems among PLH. Numerous literature sources have documented that mental health issues not only put a heavy burden on PLH but are also considered as important and common barriers to treatment adherence [6, 7]. One mental health factor that has been cited as especially important in this regard is depression [8]. In sub-Saharan Africa, depression has been shown to be the leading mental illness among PLH [7, 9, 10]. Based on self-report measures for depressive symptoms, the reported prevalence rates for depressive symptoms among PLH in sub-Saharan Africa vary quite widely, between 8 and 64% [9–12]. In Botswana, one study has reported a prevalence of depressive symptoms in women living with HIV of 48% [13]. Another, more recent study has found a prevalence rate of depressive symptoms among 291 PLH in Botswana of about 43% (Vavani et al., “Depressive symptoms among people living with HIV/AIDS in Botswana: a search for intervention targets for a coping-skills intervention,” submitted). Given the potential threat of depression to the health and well-being of PLH and to treatment adherence, it is critical to treat depressive symptoms among PLH. Unfortunately, no treatment programs specifically designed for PLH in Botswana exist. This is in part due to a mismatch between the demand for psychological services and the resources available [14]. Mental health services are provided by a limited number of psychologists and about 10 psychiatrists who serve the entire population of Botswana [15]. For a population of about 2 million, it can be assumed that the mental health services available do not reach everyone who needs them.

Until now, there is little evidence of effective intervention programs for depressive symptoms in PLH in sub-Saharan Africa. In Western countries, some intervention programs have effectively treated depressive symptoms in PLH [16–23]. Depressive symptoms were assessed through different self-report measures. The content of the programs, mostly employing techniques from the cognitive behavioral approach, include stress reduction techniques and the facilitation of adaptive coping skills (including changing of maladaptive cognitions and behaviors) [16, 24]. Some interventions occur face to face, individually, or in small groups; others are in self-help, online, or booklet format.

One such program with good results is the recently developed cognitive behavioral self-help program “Living positive with HIV”. This self-help program was developed based on findings from research on the predictors of psychological well-being in PLH. The self-help program is aimed at reducing depressive symptoms among PLH [19, 25]. It is available both online and in booklet format and is low cost. It consists of weekly lessons and can be provided with or without (minimal) coaching. Participants are expected to work on the program on a weekly basis. The Living positive with HIV program in booklet format was found to be significantly effective in reducing depressive symptoms among Dutch PLH, both in the short term and in the long term [19]. A recent randomized controlled trial (RCT) found the online version to also effectively reduce depressive symptoms among PLH [23]. The inclusion criteria for the Living positive with HIV program were being HIV positive, presenting with mild to moderate depressive symptoms, being aged 18 years and older, having adequate knowledge of the Dutch or English language, having Internet access and an email address, and being available for 8 weeks in order to complete the intervention [23].

It was argued that a similar self-help program might effectively reduce depressive symptoms in PLH in other countries, in this case Botswana, at low cost. To ensure that such a treatment program would be evidence based and would address the unique needs and circumstances of PLH in Botswana (and other sub-Saharan countries), an empirical study was performed that investigated the prevalence and risk factors of depressive symptoms, the needs of PLH, and the feasibility of an intervention program in a sample of 291 PLH in Botswana (the aforementioned study of Vavani et al.). Participants indicated a need for help with various mental health issues such as feelings of depression, feelings of anxiety, physical tension, coping with HIV, and finding new goals in life. Several coping strategies were found to be significantly related to depressive symptoms, such as rumination, positive refocusing, refocus on planning, catastrophizing, and withdrawal. Participants indicated a preference for a program in booklet format over an online program.

Based on the empirical findings and indicated needs, a self-help program with coaching in booklet format for PLH with depressive symptoms in Botswana was developed, composed of cognitive behavioral techniques, coping skills interventions, and goal adjustment training. For our purposes, goal adjustment training refers to helping patients to find realistic goals that they want to achieve and developing a concrete action plan to attain these goals.

This program will be investigated for effectiveness in the treatment of depressive symptoms among PLH. This paper describes the study protocol. By conducting an RCT, the booklet self-help program with coaching will be compared with an attention-only control condition. In addition, we will investigate treatment moderators, to study whether the program is more beneficial to some patients than to others. We will also investigate mediators of treatment, to study which mechanisms are related to treatment outcome.

## Methods/design

### Design

To evaluate the effectiveness of an intervention recently developed for PLH in Botswana, an RCT will be performed. The trial will have two conditions: an intervention group, who will receive the self-help program with coaching for depressive symptoms, and an attention-only control group, who will receive the self-help program without coaching following completion of the follow-up measurement. Random allocation of participants to the intervention or control group will be performed using a stratified random sampling technique. The sample will be stratified according to gender and treatment center. It is important to stratify by gender to ensure approximately equal proportions of males and females in the intervention and control groups. In the study by Vavani et al. that investigated intervention targets for depressive symptoms among PLH in Botswana, more females participated compared to males. Additionally, treatment centers are expected to differ with regard to the number of patients included; therefore, stratification will ensure that both the intervention and control groups in large and small treatment centers have almost the same numbers of patients in both conditions.

Random number tables will be used to allocate participants to either condition; these will be computer generated and produced by an independent researcher. Randomization will be done in permuted blocks of six. The independent researcher will enter the numbers into an Excel file which will conceal the randomization until after the participants are established in the trial. The randomization is set up such that the main researcher is unable to see treatment allocation beforehand, minimizing bias. Participants and coaches will not be blinded to allocation to conditions. Participant allocation to one of the conditions will be done after the pre-test.

The study is approved by the Health Research Development Committee of the Ministry of Health in Botswana (Ref. HPDME 13/18/1). The Standard Protocol Items: Recommendations for Interventional Trials (SPIRIT) checklist is provided as Additional file 1.

### Participants

We will recruit about 200 participants from six HIV treatment centers in Botswana. These treatment centers were selected strategically to include the two largest referral hospitals serving the northern and southern ends of Botswana. As referral hospitals, they service PLH from different parts of the country, thus giving the study an increased likelihood of recruiting participants from various backgrounds.

Recruitment of participants will be conducted using a short initial screening during their regular visits to the HIV medical staff. Further screening will be done by the researchers. The inclusion criteria for participation in the study are as follows: being HIV positive, presenting with mild to moderate depressive symptoms (that is, a score greater than 4 and less than 20 on the Patient Health Questionnaire 9 (PHQ-9) [26], aged 18 years and older, having sufficient knowledge of the Setswana or the English language, and available for the next 8 weeks to work on the intervention. The exclusion criteria are as follows: (almost) no symptoms of depression (a score of 4 or less on the PHQ-9), presenting with severe cognitive impairments (medical staff and coaches to use their clinical judgement to determine if the patient may be presenting with severe cognitive deficits), being in the first 6 months post-HIV diagnosis, and suicidality as determined by a score of > 1 on the suicide item of the PHQ-9. Those who score within the severe depressive symptoms range (≥ 20) or suicide ideation as measured by the PHQ-9 will be referred for face-to-face psychological or psychiatric treatment or to a medical doctor at the treatment center.

### Sample size

To determine the sample size needed for the RCT, we performed a power analysis using the 14th Edition of the Power Analysis and Sample Size Software (PASS) [27]. It was calculated that each group should consist of 64 participants (effect size = 0.50, alpha = 0.05, and power = 0.80). Similar studies have recorded a dropout rate of 15%; therefore, at least 75 participants should be allocated to each group. Taking into account the possibility of further dropout at follow-up, a total of 200 participants will be recruited for the RCT.

### Procedure

**Fig. 1:**
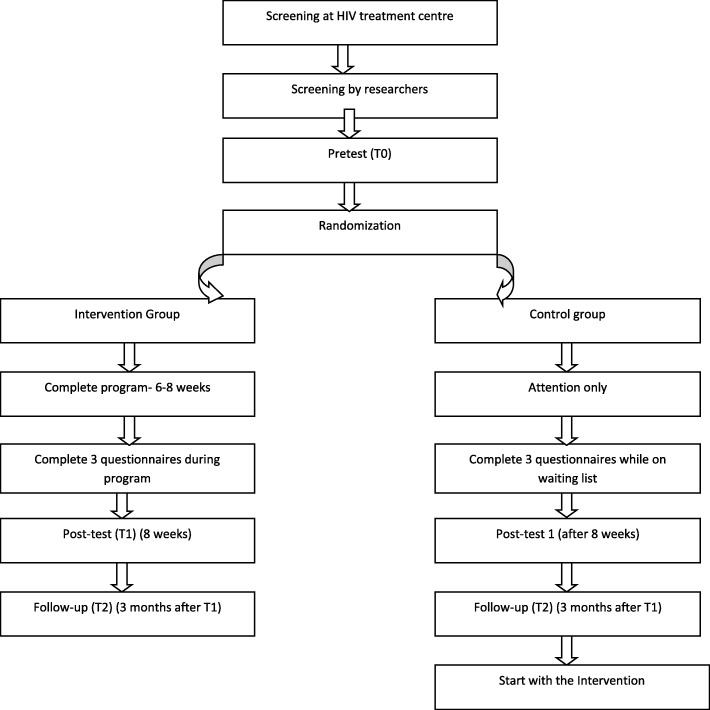
Flowchart of the study design

The study design flowchart is presented in Fig. 1. We will start by providing information about the study, screening guidelines, and forms to the treatment centers. Participants will be screened for depressive symptoms by the HIV nursing consultants and doctors during their regular check-ups at the treatment centers using the PHQ-2 questionnaire. Participants will also be screened for meeting the inclusion criteria (age 18+, HIV diagnosis longer than half a year, access to a telephone, ability to read/understand English or Setswana well, no severe cognitive limitations, such as severe forgetfulness or mental confusion, and no current treatment by a psychologist or psychiatrist at this moment) with the use of a short checklist.

In Botswana, the majority of patients who have been on treatment for more than a year visit the treatment center once every 6 months; therefore, the screening will be conducted over a period of approximately 6 months. Any patient who obtains a score > 0 on the PHQ-2 and who meets the inclusion criteria may be referred to the researchers. The HIV consultants will provide written information about the study to the patients and request for permission to provide the researchers with the patient’s contact information (telephone number or email). Following contact with the patients, the researchers will provide further information to the patients and conduct extensive screening for depressive symptoms using the PHQ-9 by telephone. Patients with a score within the mild (> 4) and moderate (< 20) ranges will be eligible for participation in the trial. These patients will then be invited to participate. Those who agree to participate will give consent by a text message to the researchers. Additionally, we will seek permission from patients to inform a medical doctor or general practitioner at the HIV treatment center about their participation in the study. Participants will then undergo a pre-test (T0). Thereafter, the participants will be randomly assigned to the intervention or the control (attention-only) group. The coaches will call all participants at the beginning of the intervention to assess for and stimulate their motivation. This will be accomplished through the use of motivational interviewing and general conversational techniques, providing information about the program, helping the patients plan for the lessons, and advising them to engage in a small activity to get started.

The intervention group will then receive the self-help treatment program with coaching by telephone, while the control group will only receive attention through a weekly telephone chat from a coach. The participants will work on the intervention for 6 weeks (with a maximum extension of 2 weeks). During the intervention, participants will be asked to answer standardized questions at three different times. At 8 weeks, all participants will complete the post-test (T1) and subsequently the follow-up measurement (T2) 3 months later. After the follow-up, participants in the control condition will receive the intervention.

The pre-test, post-test, and follow-up assessments will be done by the researchers through a standardized telephone interview; the three assessments during the intervention will be done during the coaching sessions, after the completion of lessons 1 (activation), 2 (physical relaxation), and 4 (changing negative thinking patterns). Callers will follow a standard script where they will explain who they are and the purpose of the interview. Participants will be asked questions exactly as they appear on the questionnaire. The answer categories for each section will be called out, and the participants will be asked to write them down for easy reference. Callers are allowed to repeat questions at the request of the participant.

### Study conditions: booklet self-help intervention

The booklet self-help with coaching intervention employs cognitive behavioral therapy (CBT) and stress management techniques and is based on self-regulation and stress-coping theories [28]. The content of the self-help program contains the following main components: activation, relaxation, changing maladaptive cognitions, and the attainment of new personal goals. This content is covered over six lessons to be completed in a maximum of 8 weeks and uses a combination of psycho-education, assignments, and exercises. To reduce attrition over time and to keep participants motivated, coaches will offer support and motivate participants.

The participants receive the intervention in the form of a booklet and will work on the program 1–2 h every week for 6 weeks. The first lesson is an introduction to the program and focuses on activation. Participants will be asked and encouraged to think of a small concrete new activity to perform. Throughout the program, participants will be encouraged to continue thinking about small concrete new activities to perform. Lesson 2 will be cover physical relaxation. Participants are taught to do some relaxation exercises that they continue with for the rest of the program. The third lesson will focus on changing negative thoughts. Participants are trained to recognize and change their negative thinking patterns. In lesson 4, participants will learn strategies to stop unpleasant thoughts whenever they want (for instance, by using a positive feeling). In lesson 5, participants will be helped to find new, meaningful, concrete, and time-bound life goals. They will be guided to work on attaining their goals. The last lesson will focus on achieving self-confidence to achieve their goals. Participants will learn to challenge negative thoughts which prevent them from working on their goals and gain confidence to achieve their goals. The program concludes with a summary of the important tips and tricks.

### Support from a coach

A coach will be allocated to each participant to provide support and motivation for the duration of the program. At the beginning of the program, the coaches will make telephone contact with the participants to introduce and explain technical aspects of the program and provide motivation. The coach will also schedule a weekly telephone call with the participant that will last approximately 15 min. During the telephone call, the coach will enquire about the progress of the participant and problems encountered and will offer support and motivation as well as encourage the participant to continue working on the program. Coaches follow a script to interview patients. Furthermore, coaches will be asked to record their telephone calls and also note what they discussed with the patient after every call. The audio recording and notes will be used to check if the coach follows protocol during supervision with the researchers and/psychologist. No formal psychotherapy will be provided to the participants by the coaches. During the telephone calls, the coach will provide support and motivation by enquiring about the participant’s progress, making motivational statements, and listening and providing guidance in case the participant is facing any problems. Six telephone coaching sessions will be provided in total, connected to each of the six lessons. The participant is expected to complete the program in 6 weeks, with a maximum extension to 8 weeks. If a participant has not completed the program after 8 weeks, he/she is allowed to continue with the program; however, the coaching will stop.

In the control condition, all participants will only receive minimal support in the format of six telephone calls by a coach over a period of 6–8 weeks. The weekly telephone calls between coaches and participants in the control condition will last for approximately 5 min. During this telephone call, the coaches will enquire about the participant’s well-being. Coaches will monitor the depressive symptoms of the participants and try to prevent participant dropout by helping them to explore solutions to the obstacles they face with the program, discussing the pros and cons of quitting, and continuing to make motivational remarks.

The coaches will follow guidelines on the protocol regarding provision of support and motivation. All coaches will be trained in the study procedures, and they will be expected to record each telephone call conversation and the elements used during the call such as motivation and support. Coaches will be students with a minimum of a Bachelor of Psychology degree and will have completed a clinical course during their training and been taught communication skills, interview techniques, and treatment strategies. Personal interviews will be held before coaches are appointed, and only those coaches with adequate communication skills and the capacity to provide support and motivation will be selected. Selected coaches will be provided with extra training, such as motivational interviewing skills. The researchers and a licensed psychologist will supervise the coaches. In the first month of the study, the main researcher and the coaches will hold meetings every week to discuss any challenges they are facing. Thereafter, meetings will be held every 2 weeks. Should problems arise during meetings between the coaches and the main researcher, the responsible psychologist will be contacted.

### Ethical precautions

Coaches will monitor the depressive symptoms of all participants on a weekly basis by asking how the participants are doing. When participants express severe depressive symptoms, the PHQ-9 will be administered. In case of any participant in either condition showing severe depressive symptoms (PHQ-9 > 20) or suicide ideation, the coaches will follow referral guidelines. The coaches will discuss the situation with the participant before any steps are taken. The coaches will also discuss all steps with the psychologist. The participant can be referred to a medical doctor, his/her general practitioner, or the HIV treatment center. A participant who is referred for intensive treatment to a psychologist or psychiatrist may continue with the study. However, in the analysis, the referral will be entered as a covariate to account for the influence of the treatment. These guidelines are contained in the study protocol for coaches.

### Assessments

The data will be collected at six different times during the trial (T0, three in-between assessments during the intervention, T1, and T2). Table 1 shows each stage of the study and the assessments that will be used. The initial screening (PHQ-2) will be done at the treatment centers by HIV nursing staff and doctors. At T0, T1, and T2, the researchers will conduct the assessments via a standardized telephone interview. The three in-between assessments will be conducted by the coaches by telephone.

**Table 1:** Summary of assessments for the study

Assessment	Screening HIV treatment centers *(+/− 5 min)*	Screening researchers *(+/− 10 min)*	T0: pre-test *(+/− 25 min)*	Three times during intervention/waiting period^1^ *(+/− 10 min)*	T1: post-intervention/waiting period: 6–8 weeks after T0^1^ *(+/− 20 min)*	T2: 3 months follow-up *(+/− 20 min)*
PHQ-2	X	–	–	–	–	–
PHQ-9	–	X	X	–	X	X
CES-D	–	–	X	–	X	X
Demographics and other information	–	–	X	–	X	X
Physical tension questionnaire	–	–	X	X	X	X
BADS	–	–	X	X	X	X
CERQ-short	–	–	X	X	X	X
Self-efficacy questionnaire	–	–	X	X	X	X
Goal Disengagement and Reengagement Scale	–	–	X	X	X	X
BERQ	–	–	X	–	X	X
GAD-7	–	–	X	–	X	X
PHQ-4	–	–	–	X	–	–
Life Events Scale	–	–	X	–	X	X
Motivation	–	–	X	–	–	–
Social support	–	–	X	–	X	X
Alcohol and substances	–	–	X	–	X	X
Compliance	–	–	–	X	X	–
Dropout	–	–	–	X	X	–
User satisfaction questionnaire	–	–	–	–	X	–

*PHQ-2* Patient Health Questionnaire 2, *PHQ-9* Patient Health Questionnaire 9, *CES-D* Center for Epidemiological Studies Depression Scale; BADS = Behavioral Activation for Depression Scale, *CERQ* Cognitive Emotion Regulation Questionnaire, *BERQ* Behavioral Emotion Regulation Questionnaire, *GAD-7* Generalized Anxiety Disorder 7, *PHQ-4* Patient Health Questionnaire 4

^1^Not asked to participants in the control group

To measure depressive symptom severity (primary outcome), we will use the PHQ-9 [26] and the Center for Epidemiological Studies Depression Scale (CES-D) [29]. Secondary outcomes include physical tension (physical tension questionnaire), activation (Behavioral Activation for Depression Scale (BADS) [30]), cognitive coping (Cognitive Emotion Regulation Questionnaire-short (CERQ-short) [31]), behavioral coping (Behavioral Emotion Regulation Questionnaire (BERQ) [32]), symptoms of anxiety (Generalized Anxiety Disorder 7 (GAD-7) [33]), negative life events (Life Events Scale [34]), self-efficacy (Garnefski and Kraaij, “Self-efficacy questionnaire,” unpublished), goal reengagement [35], social support, alcohol and substance abuse [36], and demographic variables. Some of the measures are single-item questions that are based on the original scales. The items were selected based on item-total correlations from our earlier research that investigated prevalence and risk factors of depressive symptoms of PLH in Botswana (Vavani et al., “Depressive symptoms among people living with HIV/AIDS in Botswana: a search for intervention targets for a coping-skills intervention,” submitted). We will also measure the participants’ motivation to start the intervention, compliance, dropout and reasons for the dropout, and user satisfaction.

Through a self-designed questionnaire, we will collect information on demographic characteristics (such as age, gender, and level of education) and clinical and psychological characteristics (such as age, depressive symptoms severity, physical health, HIV status, alcohol and substance use, motivation, and social support) as potential moderators of treatment. We will measure treatment mediators and depressive symptoms (dependent variable) during the intervention as well as during the waiting period for the control condition. The following mediator variables will be assessed in the study: physical tension (one item), activation (one item), coping (four subscales from the CERQ-short: rumination, catastrophizing, positive refocusing, and refocus on planning), self-efficacy (one item), and goal reengagement (six items). This makes it possible to examine which mechanisms precede change in the primary outcome.

On the basis of findings from our previously mentioned study that investigated the prevalence and risk factors of depressive symptoms, needs of PLH, and feasibility of an intervention program in a sample of 291 PLH in Botswana, we decided to use shortened questionnaires where possible, because respondents had great difficulty completing all the questionnaires. Where available, we used existing short versions, such as the CERQ-short, PHQ-2, and PHQ-4. Regarding the rest of the shortened questionnaires, the best items were selected based on item-total correlations in the data from the baseline study to ensure that they best fit our sample.

#### Patient Health Questionnaire 9 (PHQ-9)

The PHQ-9 will be used to measure the severity of the participants’ depressive symptoms during pre-test and post-tests [26]. It will be used for screening and for measuring change in depressive symptoms over the period of the intervention/waiting period. The questionnaire has nine items measured on a 4-point scale ranging from 0 (not at all) to 3 (nearly every day). The total score ranges from 0 to 27 with a score of ≤ 4 indicating minimal or no depressive symptoms, 5–9 mild depressive symptoms, 10–14 moderate depressive symptoms, 15–19 moderately severe depressive symptoms, and 20–27 severe depressive symptoms. A total score is obtained by adding the scores of the nine items. The scale has been widely used in measuring depressive symptoms and has good psychometric properties [26]. In addition, the PHQ-9 is often used with PLH and has been found to be highly reliable [37].

#### Patient Health Questionnaire 2 (PHQ-2)

The PHQ-2 [38] will be used to screen participants at the hospitals for depressed mood. The scale consists of the first two items of the PHQ-9 measured on a 4-point scale ranging from 0 (not at all) to 3 (nearly every day). The sum score ranges from 0 to 6 with higher scores indicating significant depressive symptoms. A total score is obtained by adding the scores of the two items. For the proposed study, any participant who scores above zero will be referred, after consent, to the study for further screening. The scale has shown good psychometric properties [38].

#### Patient Health Questionnaire 4 (PHQ-4)

The PHQ-4 [39] will be used to measure symptoms of depression and anxiety during the intervention or waiting period for the control group. The scale consists of the first two items of the PHQ-9 and the first two questions of the GAD-7 measured on a 4-point scale ranging from 0 (not at all), 1 (several days), 2 (more than half the days), to 3 (nearly every day). The sum score ranges from 0 to 12 with higher scores indicating significant depressive symptoms. A total score is obtained by adding the scores of the four items [39]. The PHQ-4 has been shown to be highly reliable in screening both depression and anxiety [39, 40].

#### Physical tension questionnaire

One item from the physical tension questionnaire (developed by Garnefski and Kraaij, unpublished) will be used to assess physical tension. The item “How often do you experience tight (or tense) feelings in your body?” was selected based on item-total correlations from our finding from a previous study that investigated the prevalence and risk factors of depressive symptoms, needs of PLH, and feasibility of an intervention program. The item is measured on a 5-point scale from 1 (always), 2 (often), 3 (sometimes), 4 (rarely), to 5 (never). A higher score indicates less physical tension.

#### Behavioral Activation for Depression Scale (BADS)

The BADS is a scale consisting of 25 items that are measured on a 7-point scale ranging from 0 (not at all) to 6 (completely). The scale is used to measure weekly changes in depression-related behaviors. It measures changes in activation, avoidance/rumination, work/school impairment, and social impairment. The scale has been reported to have good psychometric properties [30]. We will use an item from the subscale of the BADS (activation) to measure how focused the participants are on achieving certain goals and completing planned activities in the past week. The item “I was an active person and accomplished the goals I set out to do” was selected based on item-total correlations from the baseline study data and is measured on a 7-point scale ranging from 0 (not at all) to 6 (completely). Participants who score highly have greater levels of activation.

#### Cognitive Emotion Regulation Questionnaire-short version (CERQ-short)

The CERQ-short is an 18-item questionnaire used to measure cognitive coping strategies [31]. Based on our previous study findings, four subscales of the CERQ-short (rumination, catastrophizing, positive refocusing, and refocus on planning) will be used to measure the cognitive coping strategies used by the participants in relation to living with HIV [31]. The items are measured on a 5-point Likert scale ranging from a score of 1 (almost never) to a score of 5 (almost always). Each subscale has a minimum score of 2 and a maximum of 10. The subscales will be scored by adding the scores on the two items of each subscale. Higher scores on a subscale total indicate frequent use of the coping strategy. All of the subscales have been found to have good psychometric properties [31].

#### Behavioral Emotion Regulation Questionnaire (BERQ)

The BERQ is a 24-item scale that measures behaviors that PLH engage in to cope with having HIV [32]. Based on our previous study findings, behavioral coping strategies will be measured using two items from one subscale of the BERQ (withdrawal). Items were selected based on item-total correlations. Each item is measured on a 5-point Likert scale ranging from 1 (almost never) to 5 (almost always). The scores range between 0 and 10, and the total score is calculated by adding the scores of the two items of the subscale. Higher scores on the subscale total indicate frequent use of the coping strategy. All subscales of the BERQ have been found to have good internal reliability with alpha ranging from 0.86 to 0.93 [32]. In our baseline study, the alphas ranged between 0.71 and 0.84 (Table 1).

#### Generalized Anxiety Disorder 7 (GAD-7)

The GAD-7 questionnaire is used to measure the severity of generalized anxiety disorder [33]. We will use this scale to measure participants’ severity of anxiety symptoms. The scale has seven items that use a 4-point scale ranging from 0 (not at all) to 3 (nearly every day). The total scale is obtained by adding the scores of all seven items. Scores range between 0 and 21. Participants with higher scores experience more symptoms of anxiety. The scale has adequate psychometric properties [33].

#### Life Events Scale

The 17-item Negative Life Events Scale [34] was shortened to eight items and will be used to assess the experience of negative life events in the participants’ life in the past year (e.g., death of a significant other, divorce/breakup of a longstanding relationship, violence within family or partner relationship). The items were selected based on events known to correlate most strongly with depressive symptoms [41]. Furthermore, we included only life events that are relevant to our sample. The items will be measured on a 2-point scale (No or Yes), and a total score will be computed by adding all the response scores.

#### Self-efficacy

Self-efficacy will be assessed using one item from the 10-item HIV-specific self-efficacy questionnaire (Garnefski and Kraaij, “Self-efficacy questionnaire,” unpublished), according to the original questionnaire [42]. The item “I am confident that I can deal with having HIV” was selected based on item-total correlations. The item is measured on a 5-point Likert scale ranging from 1 (totally disagree) to 5 (totally agree). Higher scores indicate high self-efficacy (confidence in the ability to cope with having HIV).

#### Goal Disengagement and Reengagement Scale

Goal adjustment will be assessed using the six-item Reengagement subscale of the Goal Disengagement and Reengagement Scale [35]. The subscale was adapted to measure the participants’ ability to reengage in new goals following disengagement from goals they can no longer achieve due to the HIV (e.g., “If I have to stop pursuing an important goal in my life because I have HIV, I seek other meaningful goals”). The items are measured using a 5-point Likert scale ranging from 1 (totally disagree) to 5 (totally agree). A high score represents more goal reengagement. We included an additional item “I have already found new goals.” This item will not be used to calculate the total scores of the subscale.

#### Alcohol and substance use

A four-item questionnaire that measures the frequency of using alcohol, soft drugs, hard drugs, and sedatives in the past 3 months was developed based on the second item of the Alcohol, Smoking and Substance Involvement Screening Test (ASSIST) [36]. The ASSIST has been validated as a screening tool for alcohol and substance use cross-culturally [36]. The four-item questionnaire adapted for this study will be used to measure the frequency of alcohol and substance use (e.g., “In the past 3 months, how often have you used alcohol?”) The items will be measured on a 5-point scale ranging from 1 (never), 2 (once/twice), 3 (monthly), 4 (weekly), to 5 (daily/almost daily). Higher scores indicate frequent use of alcohol or a substance.

#### Social support

Social support will be measured by a self-designed item that measures the level of satisfaction with support received. The item “Are you satisfied with the extent to which people around you try to support you in living with HIV?” will be measured on a 5-point scale ranging from 1 (not at all) to 5 (certainly). High scores indicate satisfaction with the social support provided.

#### Demographic variables and other information

A self-designed questionnaire will be used to collect demographic information that will be used to describe the study sample. The questionnaire will collect personal information at T0 (e.g., gender, age, level of educational), past episodes of depressive and anxiety symptoms, any psychological/psychiatric treatment received for the symptoms, and physical health. Information regarding the HIV infection will also be collected through the use of a questionnaire at T0. We will ask the participants to indicate when they were diagnosed, cluster of differentiation 4 (CD4) cell count, viral load, whether they are taking ART, adherence to medication, and how satisfied they are with their HIV treatment. Questions regarding medication use for psychological problems and psychological treatment received since the beginning of the study will be asked at T0, and any changes in medication and treatment will be reported during follow-up.

#### Motivation to start with the intervention

Participants will be asked one question related to their motivation to start the intervention (“How motivated are you to start the intervention?”). Participants will rate their motivation to start the intervention on a scale from 1 (not motivated at all) to 10 (highly motivated).

#### Compliance

We will measure whether participants are adhering to the program. To assess for compliance with the program, we will ask the participants three questions to check whether they have read the information in the booklet about lessons and whether they followed the exercises in the booklet (“Which lesson(s) have you done since the last time you completed a questionnaire?”, “Did you read the text and explanation in the lessons?”, and “How often have you performed the following exercises since the last time you completed a survey?”). Participants can answer (Yes, completely), (Yes, partly), or (No, not at all) or tick lessons from the list provided for the last question. Compliance will be measured at three different points during the intervention and at post-test. Compliance will also be monitored through participant’s calls with the coach.

#### Dropout and reasons for dropout

We will take record of all participants who are no longer following the intervention and those who have no interest in completing the questionnaires. Participants are expected to complete questionnaires at three different points during the intervention/waiting period and at post-test. Participants will be asked the reason(s) for dropping out of the study.

#### User satisfaction questionnaire

A self-designed questionnaire with 31 open and close-ended questions will be used to collect information regarding participants’ satisfaction with the self-help program as well as their coach. The questionnaire will ask participants to evaluate the intervention as well as the support received from the coach assigned. Participants in the control condition will be asked 11 questions to evaluate their support from the coaches only. The responses will help in adjusting the program where necessary.

### Statistical analyses

Data will be analyzed using an intention-to-treat (ITT) analysis [43]. We will use a two-tailed alpha of 0.5 for significance testing. To investigate baseline differences between conditions, we will use chi-square and analysis of variance (ANOVA) tests for categorical and continuous variables, respectively.

Differences between groups in depressive symptoms from pre-test to post-tests will be investigated using longitudinal multilevel regression analyses (LMRAs) [44]. We will adopt a two-level model where time and group will be included as fixed effects and the slopes for time and the intercept as random effects. The between-group analysis will include the pre-test, post-test, and follow-up. To investigate the long-term effects of the intervention, we will include the pre-test, post-test, and follow-up in the within-group analysis. To estimate the effects in the model, we will employ a maximum likelihood estimation type. We will also study the differences between participants who followed the intervention to the end and those who dropped out with chi-square tests and ANOVAs.

Furthermore, Cohen’s *d* will be used to calculate within- and between-group effect sizes. Effect sizes less than 0.20 are regarded as having a small effect, 0.50 a medium effect, and 0.80 a large effect [45]. We will also explore clinically significant differences for individual change in depressive symptoms. Individual change scores of 5 or more for the PHQ-9, from pre-test to post-test and follow-up, will be regarded as clinically significant [46]. The same approach will be used to examine pre-treatment versus post-treatment changes with regard to all secondary outcomes.

We will examine moderators of the treatment outcome using LMRA. We will enter potential moderators individually. Additionally, we will employ the latent difference score model for mediation analysis [47].

## Discussion

The proposed study seeks to investigate the effectiveness of the booklet self-help program with coaching in the treatment of depressive symptoms among PLH in Botswana. In the proposed study, we will conduct an RCT and compare the booklet self-help program with coaching to an attention-only control group. We will also investigate treatment moderators and mediators. This section discusses first some strengths and thereafter some limitations of the proposed intervention program and study.

Firstly, to our knowledge, the proposed intervention program is the first treatment program designed to reduce depressive symptoms among PLH in Botswana. Therefore, if the program is found to be effective, it will help reduce the burden of depression among PLH. The program is evidence based; it was developed based on the evidence from prior research and was developed expressly for the intended population of PLH (see the aforementioned study of Vavani et al.).

Secondly, this program is of a self-help format, thus giving participants the freedom to schedule time for their lessons and allowing them to work from any place they choose. In addition, it reduces current barriers that prevent many PLH from seeking help, such as a dearth of qualified mental health care professionals in Botswana for face-to face contact and fear of stigma [13]. This way PLH who suffer from depressive symptoms throughout the country can receive help.

Thirdly, regarding the methods used in the study, we will monitor our participants’ level of depression and offer support through coaches. The support offered is likely to reduce the attrition rate. Fourth, the participants in the attention-only control group will receive the intervention after the 3 months follow-up. This means that participants will not have to wait longer than 3 months to receive treatment that they need. An additional strength of the study is that we will investigate moderators and mediators of treatment outcome, giving us valuable information regarding for whom and under what context the treatment works best. This input can eventually be used to optimize treatment and to personalize care. Lastly, we will include participants from treatment centers in different parts of the country, including the two main referral hospitals in the country. Therefore, participants from across the country have an opportunity to participate, which increases the generalizability of our study findings.

The present study also has limitations. Firstly, it is a booklet self-help program; thus, only those who can read and write can use the program. This could hamper the generalizability of our results. Secondly, we will exclude all participants whose depressive symptoms scores place them in the severely depressed range or those who indicate that they are suicidal. While these patients could still benefit from the self-help program, they require more intensive treatment. Additionally, we will conduct the follow-up 3 months following the post-test. While this period might be considered short, our argument is that it would not be ethical for participants in the control group to wait as long as 8 months before they can start the intervention. We will therefore enroll the control group to start the intervention after the 3 months follow-up.

Another limitation of the study is that, in resource-limited settings, the aspect of coaching required by this program might become an obstacle in implementing the program. Therefore, it would be necessary for future research to explore the effectiveness of the program without coaching. Furthermore, for ethical reasons, we could not include a condition without coaching to study whether coaching could influence the outcome. This is also a question that can be addressed by future research. Lastly, participants will be screened using a short screening tool (PHQ-9) for depressive symptoms, and some may argue that this tool alone is not sufficient to make a diagnosis of depression. However, the current study aims to only assess and treat depressive symptoms and not to make a diagnosis for a depressive disorder. In addition, the PHQ-9 is validated as a reliable measure for depressive symptoms severity [26, 38]. Furthermore, the presence of mild to moderate depressive symptoms is part of the inclusion criteria and not a diagnosis of a depressive disorder.

In conclusion, we aim to investigate the effectiveness of a booklet self-help with coaching intervention program for depressive symptoms among PLH. The program runs for 6 weeks (maximum 8 weeks) and includes six lessons. If the program is found to be effective, it may be implemented in Botswana. In addition, the program may be adapted and implemented in other sub-Saharan countries.

### Trial status

The booklet intervention program has been developed and translated. The materials for the RCT are ready and under translation. Recruitment of patients has not yet started.

## Additional file

Additional file 1: SPIRIT 2013 checklist: recommended items to address in a clinical trial protocol and related documents. (DOCX 45 kb)

## Data Availability

Not applicable.

## Abbreviations

ANOVA: Analysis of variance
ART: Antiretroviral therapy
ASSIST: Alcohol, Smoking and Substance Involvement Screening Test
BADS: Behavioral Activation for Depression Scale
BERQ: Behavioral Emotion Regulation Questionnaire
CBT: Cognitive behavioral therapy
CD4: Cluster of differentiation 4
CERQ: Cognitive Emotion Regulation Questionnaire
CES-D: Center for Epidemiological Studies Depression Scale
GAD-7: Generalized Anxiety Disorder 7
ITT: Intention-to-treat
LMRA: Longitudinal multilevel regression analysis
PASS: Power Analysis and Sample Size Software
PHQ-2: Patient Health Questionnaire 2
PHQ-4: Patient Health Questionnaire 4
PHQ-9: Patient Health Questionnaire 9
PLH: People living with HIV
PMTCT: Prevention of mother-to-child transmission
